# Assessing the feasibility of heart rate variability as an objective indicator of anxiety in older adults with dementia living in care homes

**DOI:** 10.1186/s13104-021-05458-2

**Published:** 2021-02-05

**Authors:** Milena A. Quinci, Arlene J. Astell

**Affiliations:** 1 School of Psychology & Clinical Sciences, University of Reading, Reading, RG6 7BE UK; 2 Centre for Cognitive & Brain Health, Northeastern University, Boston, MA USA; 3 Department of Occupational Sciences & Occupational Therapy and Department of Psychiatry, University of Toronto, Toronto, Canada; 4 KITE, University Health Network, Toronto, Canada

**Keywords:** Dementia, Anxiety, heart rate variability, long-term care

## Abstract

**Objective:**

Anxiety is reportedly prevalent in older adults with dementia living in care homes and, within this population, is most often assessed through caregiver reports. Heart rate variability (HRV) is a physiological indicator of autonomic function, whereby reduced vagally-mediated HRV is associated with a variety of anxiety symptoms and disorders. This study evaluates the feasibility of collecting HRV data within this population, presents HRV data for older adults with dementia living in a care home, and examines HRV in the context of self-reported anxiety. These data were collected during a larger study examining an exercise intervention.

**Results:**

HRV data, in the form of log-transformed root mean square of the successive differences (lnRMSSD), were in line with transformed data from previous research. These data provide a promising direction for the use of wrist-worn devices in future HRV research with people living with dementia in care homes.

## Introduction

Anxiety disorders are characterized by excessive worry, restlessness, and irritability [1]. Physiological hallmarks include increased heart rate and respiratory frequency and reduced parasympathetic activity [2]. Anxiety disorders are common among people with dementia [3], with a high reported prevalence within the care home population [4]. Caregiver observations or reports are commonly used to assess anxiety in people with dementia but have a low correlation with self-reported symptoms [5].

Heart rate variability (HRV), a physiological index used to assess autonomic nervous system (ANS) activity, measures the variability in time between two consecutive heartbeats, also referred to as RR-intervals [6]. Traditionally assessed using an electrocardiogram (ECG), wearable devices such as chest straps and wrist-worn monitors are also gaining popularity [7]. HRV data are most commonly analysed using time-domain and frequency-domain measures. Time-domain measures, which quantify the variability in RR-intervals, include standard deviation of all R-R intervals (SDNN), percentage of consecutive regular sinus RR-intervals over 50 ms (pNN50), and root mean square of the successive differences (RMSSD) [8]. Frequency-domain measures, which quantify oscillatory components into different frequency bands [9], include ultra-low frequency (ULF), very-low frequency (VLF), low frequency (LF), high frequency (HF), and low frequency/high frequency ratio (LF/HF) [8]. Each measure of HRV is associated with different autonomic branches, with RMSSD and HF indicating parasympathetic activity [6]. Lower parasympathetic, or vagally-mediated, HRV has been associated with a variety of anxiety symptoms and disorders [10].

The overall goal of this novel project is to present HRV data for older adults with dementia, specifically living in a care home. More specifically, this project aims to (1) evaluate the feasibility of collecting HRV data within the dementia care home population; (2) investigate autonomic functioning, as measured through HRV, in the older adults with dementia living in a care home; and (3) discuss these HRV results in the context of self-reported anxiety.

## Main text

### Methods

Ten participants (3 males, 7 females) with a mean age of 90 (*SD* = 5.80; Table 1) were recruited from a specialized dementia long-term care home. Age, dementia diagnosis, mental health diagnoses (if any), and current medications were extracted from records. Participants’ cognitive functioning was assessed using the Montreal Cognitive Assessment (MoCA) [11], which confirmed that all participants had cognitive impairments (indicated by a MoCA score of < 26/30). The Hospital Anxiety and Depression Scale anxiety subset (HADS-A) was used to assess participants’ anxiety, with a score of greater than 10/21 considered abnormal as per the authors’ instructions [12]. This measure was chosen as it is a short assessment used previously to evaluate self-reported anxiety in PWD [13]. HRV data were collected using the Zoom HRV wrist-worn monitor. A wrist monitor was used as it is completely non-invasive and easily portable [7]. The data were saved to the Elite HRV iPhone application. Kubios HRV Standard software was used for the analysis of the RR-interval data.

**Table 1:** Participant demographic information

Participant ID	Age	Dementia diagnosis	Mental health diagnoses (if any)	MoCA score (out of 30)
P1	97	Dementia	–	4
P2	88	Dementia	Anxiety	0
P3	96	Alzheimer’s	–	15
P4	96	Alzheimer’s	–	8
P5	88	Dementia	–	6
P7	95	Dementia	–	7
P8	85	Mixed and Vascular Dementia	Depression	10
P9	88	Alzheimer’s and Vascular Dementia	–	9
P10	87	Dementia	–	3
P11	80	Vascular Dementia	–	20
Mean (SD)	90 (5.80)	–	–	8.2 (5.54)

Data were collected at the care home, in either a small library or lounge. The wrist-worn monitor was placed on participants’ non-dominant wrist to reduce the residual effects of daily activities frequently performed using the dominant hand [14] on the data. Participants were asked to sit in an upright position, keep their eyes open, and remain still and quiet during this portion of data collection. Despite these instructions, most participants moved occasionally or engaged in conversation with a staff member or volunteer. RR-interval recordings were taken after participants had been seated for approximately one minute. Two recordings of RR-interval data were collected from each participant.

Following HRV data collection, the Hospital Anxiety and Depression Scale (HADS) statements and response options were read aloud to the participants as many of them had poor eyesight which interfered with their ability to read the statements. Participants were asked to articulate their response to each statement aloud. Since the HADS assessment contained statements regarding sensitive subject matter, special attention was paid to the participants’ well-being during the administration of this assessment. Subsequently, a second researcher administered the MoCA as per the administration guidelines.

### Data analysis

Two shorter epochs, rather than one longer one, were collected due to the limitation of the Zoom HRV device and to minimize non-stationarities in the data [15]. These epochs ranged from 65 to 156 s (*M* = 126.1). Prior to analysis, a few of the epochs were shortened due to signal loss. The range of epoch times remained the same, but the mean differed slightly (*M* = 125.5).

Artefact correction was performed using threshold-based artefact correction. Each epoch was corrected using a different threshold strength, as there was variation in the quality of the RR-interval data between different participants and epochs. The lowest strength threshold that allowed for the identification and correction of the most artefacts was chosen. In a majority of the epochs, the percentage of beats corrected did not exceed 5% [16]. Some epochs required more rigorous corrections. Just over 5% of beats were corrected in two of the epochs [17]. The artefact corrected data were detrended using the smoothn priors method [18] with a lambda value of 500 [16]. The data were then analysed in the time domain to produce RMSSD values. The two RMSSD values per participant, in units of milliseconds (ms), were prorated to equal time intervals of 60 s to correct for differing epoch durations [19] and averaged so each participant had one RMSSD value.

The Shapiro–Wilk test was run for the averaged, prorated RMSSD values to ensure normal distribution. The data were not normally distributed (*p* < 0.05). To correct for this, the data were transformed using the natural log function (ln) [6]. The log transformed RMSSD (lnRMSSD) values were normally distributed, *W*(10) = 0.927, *p* > 0.05.

## Results

To our knowledge, there are no data reporting average HRV for the dementia care home population to use as a direct means of comparison. Table 2 summates data from past studies investigating time-domain HRV in older adults of a variety of ages and physical capabilities. These indicate a range for mean log transformed RMSSD data for older adults between 3.05 and 3.30. The mean lnRMSSD from the participants in this study was 3.17 (Table 3).

**Table 2:** Articles reporting time-domain analysed HRV for older adults

Author and Date	N	Population	Dementia	Age range or Mean age (years)	HRV recording device	RMSSD (ms)	lnRMSSD (ms)
Albinet et al. (2010) [20]	24	Sedentary older adults	No	70.7	Polar Wearlink® Wind transmitter belt	23.7–26.9	3.05–3.09
Almeida-Santos et al. (2016) [21]	1743	Functionally independent adults with satisfactory	No	40–100	ECG	30.22	3.3
	169	Cognition		≥ 80		33	NR
de Vilhena Toledo and Junqueira (2008) [22]	22	Older adults with probable Alzheimer’s disease	Yes	60–87	ECG	14.0	NR
	24	Healthy older adults	No	60–91		17.6	NR
Kasanuki et al. (2015) [23]	30	Patients with probable dementia with Lewy bodies	Probable		ECG	14.2	NR
	3	Patients with Alzheimer’s disease	Not specified	79.6		27.9	NR
	20	Healthy elderly adults	No	77.2		25.4	NR
Kurita et al. (2006) [24]	12	Elderly adults hospitalized for cerebral vascular disease and dementia	Yes	88	ECG	19.4	NR
Zulfiqar et al. (2010) [25]	20	Health older adults	No	80–99	Holter monitor	30	NR

**Table 3:** Participant HRV and self-reported anxiety

N = 10			
Participant ID	RMSSD	lnRMSSD	HADS-A
P1	13.21	2.58	6
P2	8.70	2.16	12
P3	92.43	4.53	4
P4	11.23	2.42	3
P5	5.14	1.64	2
P7	31.31	3.44	4
P8	98.99	4.59	4
P9	78.57	4.36	2
P10	25.32	3.23	7
P11	15.98	2.77	9
M(SD)	38.09 (36.95)	3.17 (1.04)	5.30 (3.23)

## Discussion

This study aimed to assess the feasibility of collecting HRV data in the dementia care home population. The few existing studies exploring HRV in people with dementia found that this population exhibits reduced HRV [22–24, 26]. Reduced HRV has also been found in older people without dementia who exhibit cognitive impairments [8]. Given that reduced autonomic functioning is associated with dementia, it is important to identify a normal HRV range for older adults with cognitive impairments. Our log-transformed mean RMSSD data fell between the reported mean lnRMSSD of 3.05 by Albinet et al. [20] and 3.30 by Almeida-Santos et al.[21], suggesting that our participants exhibited a similar level of parasympathetic control to older adults without cognitive impairments described in previous studies. Data presented in this paper adds to the current body of literature assessing autonomic functioning in older adults with dementia.

This study also investigated the use of wrist-worn devices, which have been previously used to explore autonomic functioning in older adults with dementia over the course of their daily routine [27, 28]. Despite the promising implications of wrist-worn devices, there is limited literature that explores the use of these devices in a clinical context [7]. This paper may be among the first to report time-domain analysed HRV values collected using a wrist-worn device for PWD. Thus, our data adds to the growing body of literature exploring the use of wrist-worn devices for clinical purposes.

Based on past literature indicating a high level of anxiety (up to 70%) in people with dementia [3], it was expected that the majority of participants in the current study would have anxiety. However, only one participant received a HADS-A score between eight and ten, which is classified as borderline abnormal, and one participant received a score that was considered abnormal (12). This same participant was the only one who had a previous diagnosis of anxiety in her medical records. This suggests that, though self-report measures of anxiety are often overlooked for caregiver reports [3], these measures may provide a reliable assessment of anxiety within the dementia care home population. These findings indicate a need for the prevalence of anxiety within this population to continue to be evaluated. Given the relationship between HRV and anxiety described in previous literature [10], future studies should explore the relationship between anxiety and HRV, collected using wrist-worn devices, to see if HRV could be used as an objective indicator of the prevalence of anxiety in the population living in care homes with dementia.

## Limitations

A small sample size could have hindered the detection of any significant findings of a relationship between anxiety and HRV. This is a common problem for HRV studies, which are often underpowered [6]. A second limitation was participants’ prescribed medications. Antidepressant, antipsychotic, antihypertensive, and cardio-related medications have been said to affect HRV. All participants were on at least one medication, and seven were taking at least one medication that fell into one of the categories indicated by Laborde et al. (2017) as exclusionary. A third limitation was the short epochs of HRV data that were able to be collected by the Zoom HRV per each recording. The device was chosen as it was the least invasive device available that had the ability to measure and export HRV data and had been used in other studies [29]. Although, this is a sufficient amount of data to accurately assess RMSSD [6], all the epochs were of different lengths as the device stops recording when the signal is lost, making it impossible to control the length of each epoch. Future studies should continue to evaluate different wrist-worn monitors that are able to record more consistent epochs of RR-interval data. Overall the study demonstrated the feasibility of collecting HRV data in a dementia care home environment in a manner that was acceptable to the residents. This shows great promise for future research studies and clinical practice to accurately detect the presence of anxiety in this population and intervene to reduce symptoms and improve wellbeing of people living with dementia in care homes.

## Data Availability

The datasets used and/or analysed during the current study are available from the corresponding author on reasonable request.

## Abbreviations

ANS: Autonomic Nervous System
HADS: Hospital Anxiety and Depression Scale
HF: High Frequency
HRV: Heart Rate Variability
LF: Low Frequency
LF/HF: Low frequency/high frequency ratio
lnRMSSD: Log transformed RMSSD
MoCA: Montreal Cognitive Assessment
RMSSD: Root mean square of the successive differences
SDNN: Standard Deviation of R-R intervals
R-R interval: Variability in time between two consecutive heartbeats
ULF: Ultra-low frequency
VLF: Very-low frequency low frequency
